# Debunking the myth of using “quiet” in clinical departments: an integrative overview of available literature

**DOI:** 10.1016/j.amsu.2022.104792

**Published:** 2022-10-06

**Authors:** Tungki Pratama Umar, Nityanand Jain

**Affiliations:** a Faculty of Medicine, Sriwijaya University, Palembang, Indonesia; b Faculty of Medicine, Riga Stradiņš University, Dzirciema Street 16, Riga, LV-1007, Latvia

**Keywords:** Quiet, Clinical department, Myths, Peaceful, Overcrowding, Evidence

Commonly referred to as the “art of treatment”, medical practice is an evidence-backed and peer-critiqued field of science. However, there exists several myths and misconceptions in current practice that seem to stem from personal idiosyncrasies and have been passed on over the years (telling and retelling) due to unavailability of logical explanation or credible evidence. Such idiosyncrasies provide the believers a sense of meaning, justification, and security [1].

In emergency medicine, there exists many such misconceptions including prevalence of “non-urgent cases”, and the influence of using words like “quiet” and “peaceful” on the levels of the crowding in the department [2,3]. In the present study, henceforth, we sought to investigate the latest evidence supporting the relationship between using the word “quiet” and its impact on clinical department overcrowding.

A PubMed database search from January 2015 to July 2022 for modern and current evidence-based English language articles (randomized controlled trials on human participants only) was conducted that investigated the relationship between the use of the word “quiet” and its corresponding impact on the perception of overcrowding in various clinical departments. Randomized Controlled Trials (RCTs) were included since they are considered the gold standard for comparing the effectiveness of various intervention strategies, given their ability to prevent bias in clinical trial design whilst controlling confounding factors and enhancing the efficacy of the statistical tests [4].

We were able to retrieve five such open-access full-text articles [2,3,[5], [6], [7]], which we then benchmarked against the Joanna Briggs Institute's (JBI) 2017 Critical Appraisal checklist for Randomized Controlled Trials (available at https://jbi.global/sites/default/files/2019-05/JBI_RCTs_Appraisal_tool2017_0.pdf; accessed 25th June 2022) for assessing the quality of the data reported (Table 1).

**Table 1:** JBI's Critical Appraisal checklist for RCTs.

*Criteria*	*Kuriyama* et al.*, 2016* [7]	*Lamb* et al.*, 2017* [5]	*Brookfield* et al.*, 2019* [4]	*Go* et al.*, 2022* [8]	*Geller* et al.*, 2022* [9]
Was true randomization used for assignment of participants to treatment groups?	Yes	Yes	Yes	Yes	Yes
Was allocation to treatment groups concealed?	Yes	No	No	No	No
Were treatment groups similar at the baseline?	Yes	Yes	Yes	Yes	Yes
Were participants blind to treatment assignment?	Yes	Yes	No	Yes	Yes
Were those delivering treatment blind to treatment assignment?	No	No	No	No	No
Were outcomes assessors blind to treatment assignment?	No	No	No	No	No
Were treatment groups treated identically other than the intervention ofinterest?	Yes	Yes	Yes	Yes	Yes
Was follow up complete and if not, were differences between groups interms of their follow up adequately described and analysed?	N/A	N/A	N/A	N/A	N/A
Were participants analysed in the groups to which they were randomized?	N/A	N/A	N/A	N/A	N/A
Were outcomes measured in the same way for treatment groups?	Yes	Yes	Yes	Yes	Yes
Were outcomes measured in a reliable way?	Yes	Yes	Yes	Yes	Yes
Was appropriate statistical analysis used?	Yes	No	Yes	Yes	No
Was the trial design appropriate, and any deviations from the standard RCT design (individual randomization, parallel groups) accounted for in the conduct and analysis of the trial	Yes	Yes	Yes	Yes	Yes
***Additional Comments***					
Country of Trial	Japan	United Kingdom	United Kingdom	United States of America	United States of America
Department of Investigation	Emergency Department	Multi-centre	Clinical Microbiology	ENT Department	Emergency Department
Number of days investigated	347 shifts in two separate trials	42 shifts	61 days	80 shifts	47 shifts
Method of Randomization	Computerized random number generator	Coin toss	Big stick procedure	Microsoft® Excel 2016	Online randomizer
Measurement of outcome of interest (workload reduction)	Number of patient, stress levels, mealtime duration, fatigue, number of all admissions during their shift, and number of all ambulatory and transferred patients who visited the ED during their shift.	Number of referrals on a day	Number of visits and phone calls on a day	Number of consults per day and subjective survey on the perception of workload	Number of patient visits and workload (VAS scale)
Strengths of the study design	The only registered trial	Multi-centre study design	Inclusion of possibly affecting day: full moon, solstices or equinoxes, or a Friday on the 13th day of the month	Matched groups, Ethics approval reported	Ethics approval reported
Limitations of the study design	Single centre, single blind, non-matched, no ethics reported	No ethics reported, non-matching amount of day, no weekday vs. weekend comparison, multi-centre but not evenly distributed, single blind	Non-matching amount of day, non-stratified randomization, no ethics reported, convenience sampling, single centre	Loss of data (8/80, 10%), depends on registrar note	Convenience sampling, multiple collection, exact participants not mentioned, subjective, no validation of VAS, single centre
Use of appropriate reporting guidelines	–	–	SPIRIT guidelines	–	CONSORT guidelines
Main Conclusion	No significant difference between control and intervention group	Significant differences between intervention and control group (busier in quiet group)	No significant difference between control and intervention group	No significant difference between control and intervention group	No significant difference between control and intervention group
**Perceived Strength of Evidence**	Moderate	Low	Low	Low	Low

Note: N/A = Not applicable.

Abbreviations: CONSORT = Consolidated Standards of Reporting Trials; ENT = Ear, Nose, and Throat; SPIRIT = Standard Protocol Items: Recommendations for Interventional Trials; VAS = Visual Analog Scale.

Most studies (four out of five) reported that usage of the term “quiet” was not associated with increased patient volumes or the perception of increased patient volumes. Although the findings may provide new evidence to debunk the myth and focus physicians' attention on their work, a lack of rigorous methodology and lapses in adherence were identified in the included studies. The perceived strength of the available evidence is low for most studies (Table 1).

Systematic issues confounding all the investigated studies related to the participants' repeated measures, which may impact the study's conclusion, and the lack of a random allocation procedure because the participants may be in the intervention or control groups simultaneously. Furthermore, as the intervention is measured repeatedly on the same participants, blinding to the participants may fail, mainly due to the intervention of using the term “quiet” being a common myth for emergency practitioners [8]. As a result, blinding would not occur continuously throughout the study. To overcome this issue only Kuriyama et al. conducted sensitivity analysis and found no significant differences in the results of primary measured outcomes for both of their trials [5]. Another factor affecting the validity of the investigated studies is the non-matching of weekends and weekdays.

Baseline variations arising from personal believes of the investigated staff, their experience, knowledge on the topic, age, gender, and seniority have also been largely ignored in the reported studies. Lack of adherence to presenting proper reporting guidelines in three of the five studies also questions the reliability of these trials. Furthermore, when designing a randomized controlled trial, the convenience sampling method described in the studies is still debatable. Additionally, as the studied shifts/days are less than 100 in most of the studies, the different randomization methods employed by the studies might seem to be unsuitable as it can lead to unequal sample size between each group [9].

Finally, as previously indicated, four studies reported inconsequential findings, in contrast to one study that demonstrated significant results of using the phrase “quiet” in the multicentre orthopaedic emergency setting. However, the abundance of red flags in this study greatly raises doubts about how the findings might be interpreted. Firstly, utilizing a coin flip approach for allocation concealment is not ideal because it might be redone at the employee's request and could result in an imbalance in baseline characteristics. As a result, it needs to be rethought before replication in a small-scale clinical trial [10]. Secondly, given the lack of valid excuse for the selection of shifts and the determination of sample size, the slightly significant p-value of 0.04 raises serious concerns about “p-hacking,” causing the underreporting of real effect sizes in published research.

In real-life scenarios, however, several tangible factors contribute to clinical department overcrowding - external (like patient demographics, accessibility, and devastating phenomenon) and internal factors (personnel availability, admission system, patient flow management, and hospital space). Overcrowding caused by these phenomena can have a significant impact on the economics of the hospital, psychology of the patients, and quality-of-care provided by the personnel (Fig. 1). Based on our review of the studied literature, there is no evidence that usage of any words or expectations of future clinical workload in emergency settings, including the use of “quiet” words, may influence departmental overcrowding. As a result, it was not included in our proposed cause-effect model.

**Fig. 1:**
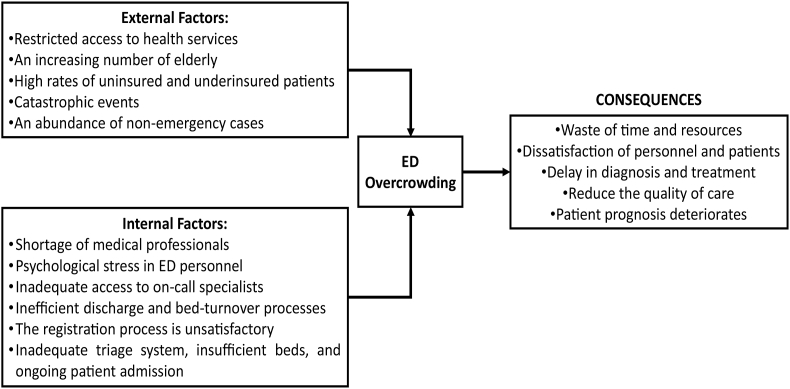
Cause-effect model (CEM) of factors influencing overcrowding in the emergency departments in the hospital.

Moving forward, future study designs should try to eliminate as many of the highlighted limitations of the previous studies, especially by conducting multicentre trials (which can offer larger sample sizes and larger observation periods for more generalizable findings). The published studies, despite their limitations, repetitively provide empirical support for the absence of an effect. Adequate reporting and publication of negative findings are informative because it allows researchers to deny a theory. Consequently, sufficient publication of non-significant findings makes scientific literature more complete, allowing for a more accurate assessment of a scientific work's replicability.

## Ethical approval

Not Applicable.

## Source of funfing

None.

## Author contribution

TPU and NJ conceptualized the report, whilst both authors were involved in the data collection and preparation of the manuscript. Both authors have read and agreed to the final version of the report for publication.

## Trail registry number

None.

## Garantor

Both Authors.

## Declaration of competing interest

None declared.
